# Influence of colour and age on the thickness and medullation characteristics of wool Huacaya alpacas maintained in Poland

**DOI:** 10.5194/aab-67-401-2024

**Published:** 2024-08-16

**Authors:** Ewa Kuźnicka, Katarzyna Stempke, Aurelia Radzik-Rant, Witold Rant

**Affiliations:** Institute of Animal Sciences, Warsaw University of Life Sciences-SGGW, Ciszewskiego St. 8, 02-786 Warsaw, Poland

## Abstract

The aim of this study was to analyse the thickness and medullation of Huacaya alpaca fibre depending on the colour of the wool and the age of the animal. The analyses were conducted on 30 females with ages of under 1 year (12), from 1 to 4 years (6), and over 4 years (12). There were individuals with white (12) and brown (18) wool in all age groups. The animals were maintained in the same conditions; they were fed hay and concentrate feed throughout the whole year, with permanent access to water, and in the spring and summer periods, they were also fed grass. The fibre diameter and medullation were measured using a projection microscope according to the IWTO-8-2011 standard. The analysis showed no significant effect of the wool colour variety on the average fibre diameter and the degree of medullation, in contrast to the age. The average fibre diameter and fibre medullation percentage were lower in alpacas up to 1 year old ( P<0.05 ) than in alpacas aged 1 to 4 years and over 4 years old. All types of fibres (non-medullated, with continuous and discontinuous medullas) were present in the wool of the examined alpacas, regardless of the thickness and/or colour of the wool and the age of the animals.

## 1 Introduction

The world alpaca (*Vicugna pacos*) population is estimated to be approximately 4 million, of which 80 % are bred in the Andean area of Peru (Quispe et al., 2013). The rest of the population is mainly located in Bolivia and Chile. Due to the high adaptability to difficult environmental conditions, relatively low nutritional requirements, and the great interest in alpaca wool, herds of these animals can be found all over the world (McGregor and Butler, 2004; Wuliji et al., 2000; Krajewska-Wędzina et al., 2020). The presence of alpacas is also recorded in Poland, and including them as farm animals has significantly increased the interest of breeders in keeping these animals (Radzik-Rant et al., 2021; Czaplicki, 2012). The main reason for the dissemination of alpaca breeding across different continents is their wool, with a fibre thickness of 15 to 30 µ m (Quispe et al., 2013; Alfonso et al., 2008; Lupton et al., 2006; Radzik-Rant et al., 2018).

The coat of the alpaca is characterized by uniformity of the wool, although it shows a relatively large variability in terms of thickness and a significant presence of medullated fibres. The presence of the medulla occurs mostly in fibres of larger diameter, but it is also often present in thinner fibres. Many studies address the problem of needing to select alpacas with the intention of not only reducing the fibre diameter but also eliminating the degree of fibre medullation (Cruz et al., 2019; Pinares et al., 2018; Gutiérrez et al., 2011; Gutiérrez et al., 2014). Alpaca fibre is considered to be extremely delicate and is characterized by excellent thermal insulation, which may be due to the presence of the medulla; however, the medullation also increases the discomfort of using finished products compared to products made of other fibres (Süpüren et al., 2015; Valbonesi et al., 2010; Frank et al., 2017). Nevertheless, the textile industry considers alpaca wool to be a unique raw material, and clothing made of it is considered to be a luxury (Wang et al., 2005).

The thickness of the fibres and the degree of their medullation depend on many factors, including the colour of the wool, as well as the age, sex, physiological state, and living conditions of the animal (McGregor, 2006; Lupton et al., 2006; Frank et al., 2006; Braga et al., 2007; Montes et al., 2008; Cruz et al., 2017). The impacts of some of these factors are better understood, while others are still unclear. Many authors believe that the white wools are finer and contain less medullated fibres than other coloured varieties and that wool from males is coarser than that from females (McGregor, 2006; Cruz et al., 2017; Radzik-Rant and Wiercińska, 2021). According to Lupton et al. (2006) and Wuliji (2017), both the thickness of the fibres and the degree of medullation of the wool increase with the age. In turn, the lack of relationship between thickness and colour is indicated by Wuliji et al. (2000) and Wurzinger (2006), while Frank et al. (2006) determined a smaller fibre diameter in dark wool. Although, due to the requirements of the textile industry, most alpacas have a white coat, there will also be an increase in interest in colour varieties. This is associated with an increase in allergies among customers, and thanks to the natural colour of wool, patterned fabrics can be obtained without the need to dye them (Brenes et al., 2001).

Due to the variety of research results regarding the influence of various factors on the characteristics of alpaca wool, there is still a need to conduct research, especially on animals kept in conditions other than South American ones. Therefore, the aim of the study was to compare the thickness and fibre medullation of alpacas' white and coloured wools obtained from the animals of different ages kept in Poland.

## 2 Materials and methods

### 2.1 Animals

The investigation was carried out in central Poland, with a mean annual temperature of 7.5 °C and mean annual precipitation of 528 mm.

For the study, we selected 30 female alpacas of the Huacaya herd, aged up to 1 year (12 animals), over 1 to 4 years (6 animals), and over 4 years (12 animals). There were animals with white hair cover (12 animals) and coloured hair cover (18 animals) in all age groups (Table 1).

**Table 1:** The number of alpacas of different colour varieties within the different age groups.

Colour variety	Age up	Age from 1	Age over	Total
	to 1 year	to 4 years	4 years	
White	3	3	6	12
Coloured	9	3	6	18
Total	12	6	12	30

The animals were kept in a semi-open shed with access to the paddock. The whole year's feeding was based on hay and concentrate feed, offered at 250 g d ${}^{-1}$ per head. The concentrate was composed of 18.5 % crude protein, 3.4 % crude fat, and 6.5 MJ of energy. From spring to autumn, the animals were grazed with permanent access to water.

### 2.2 Samples

The analysed wool samples were taken during shearing from the most representative zone (Aylan-Parker and McGregor, 2002), the middle of the left side of the trunk and the middle part of the 10th rib midway between the back line and belly. The animals were sheared once a year. The samples were sealed in plastic bags and stored for later analysis in the laboratory.

After being cleaned by hand using “grey” soap and dried at room temperature for 24 h, wool samples were subjected to fibre diameter and medullation using a projection microscope according to the IWTO-8-2011 standard. At least 600 fibres were measured in each sample. In total, 18 000 fibres were analysed. The choice of the measurement method was dictated by the possibility of determining the type of medulla.

The mean fibre diameter (MFD), the standard deviation of the mean fibre diameter (SD), and the coefficient of variation of the mean fibre diameter (CV) were determined. Each fibre was classified according to the category of the medulla into non-medullated, discontinuously medullated, and continuously medullated fibres. The frequency of medullated fibres (FMs) was estimated as a percentage of the number of fibres measured in the sample considering the proportion of all types of fibres presented above.

**Figure 1:**
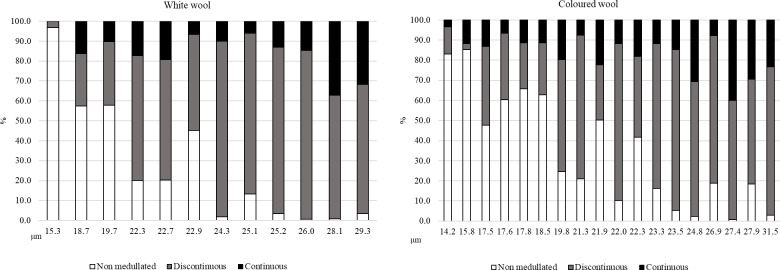
The share of different types of fibres in the tested samples of alpaca wool depending on colour variety.

### 2.3 Statistical analysis

A statistical analysis of the fibre diameter and medullation was performed using SPSS 23.0 software (2016) based on a linear model that included the effect of age, colour variety, and the interaction between age and colour variety. All effects were tested against the residual middle squares to determine the level of significance. P values lower than 0.05 were considered to be statistically significant. The results are presented as the mean for each trait along with the standard deviation ( ± SD).

## 3 Results

In the herd under study, no significant differences were found in the mean fibre diameter (MFD) of the hair coats of white and coloured alpacas. The thickness of the white alpacas' wool ranged from 15.25 to 29.28 µ m, and that of the coloured ones' wool ranged from 14.22 to 31.45 µ m. Only one coloured individual had a wool thickness exceeding 30 µ m. The two tested colour varieties of wool did not differ significantly in terms of the standard deviation (SD) of the mean fibre diameter and the coefficient of variation (CV) of the MFD. Despite the lack of statistical differences, white wool was thinner than coloured wool, but it was characterized by greater variability of this feature (Table 2).

**Table 2:** The wool thickness and medullation features depending on colour variety (mean ± SD).

Item	White wool	Coloured wool	P value
Mean fibre diameter (MFD) ( µ m)	22.38 ± 1.15	23.42 ± 1.09	0.482
Standard deviation (SD) of MFD	6.23 ± 0.91	5.68 ± 0.22	0.551
Coefficient of variation (CV) of MFD (%)	29.00 ± 4.33	25.45 ± 2.24	0.432
Non-medullated fibres (%)	36.03 ± 8.92	26.49 ± 6.67	0.370
Medullated fibres (%)	63.97 ± 8.92	73.52 ± 6.67	0.370
Continuous medullated fibres (%)	14.07 ± 3.04	18.04 ± 2.27	0.349
Discontinuous medullated fibres (%)	49.90 ± 7.62	55.48 ± 5.73	0.555

Although the differences between the colour varieties in terms of medullation characteristics were not statistically confirmed, the frequency of medullated fibres (FMs) in white wools was slightly lower (63.97 % vs. 73.52 %) compared to in coloured wools (Table 3). A higher content of non-medullated fibres was observed in the thinner wools of both colour varieties; wools with a larger mean fibre diameter were characterized by a higher content of medullated fibres, among which fibres with discontinuous medullation prevailed (Fig. 1).

**Figure 2:**
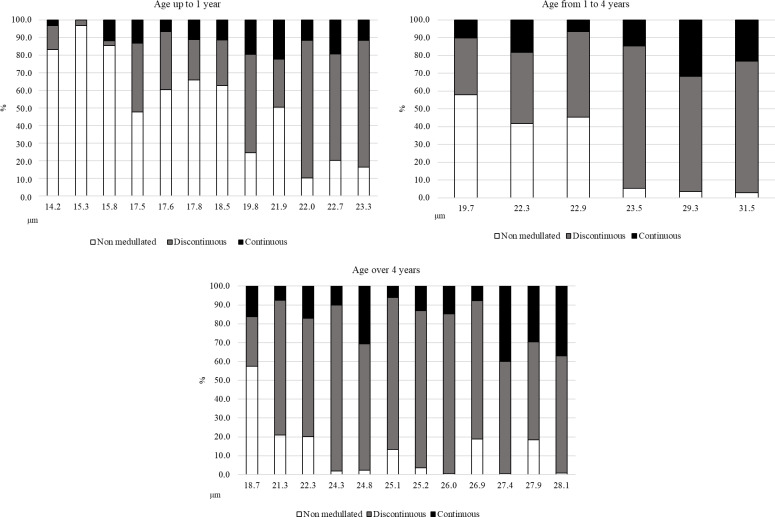
The share of different types of fibres in the tested samples of alpaca wool depending on age.

Analysing the parameter fibre thickness depending on age indicated that the average fibre diameter was lower in alpacas up to 1 year old ( P<0.05 ) than in alpacas aged 1 to 4 years and over 4 years old. The tested wool in all age groups did not differ in terms of deviation from the mean thickness (SD), while the coefficient of variation (CV) was higher ( P<0.05 ) in the wool of alpacas aged up to 1 year old than in other tested age groups (Table 3). The thickness of the up-to-1-year alpacas' wool ranged from 14.22 to 23.32 µ m, that of alpacas aged 1 to 4 years ranged from 19.69 to 31.45 µ m, and that of alpacas aged over 4 years old ranged from 18.72 to 28.12 µ m.

**Table 3:** The wool thickness and medullation features depending on the alpacas' ages (mean ± SD).

Item	Age up to 1 year	Age from 1 to 4 years	Age over 4 years
Mean fibre diameter (MFD) ( µ m)	18.90 ${}^{a}$ ± 1.33	24.86 ${}^{b}$ ± 1.40	24.95 ${}^{b}$ ± 1.01
Standard deviation (SD) of MFD	6.80 ± 0.83	5.29 ± 0.87	5.77 ± 0.63
Coefficient of variation (CV) of MFD (%)	36.27 ${}^{a}$ ± 4.07	22.12 ${}^{b}$ ± 4.29	23.28 ${}^{b}$ ± 3.08
Non-medullated fibres (%)	54.61 ${}^{a}$ ± 9.57	26.07 ± 10.09	13.09 ${}^{b}$ ± 7.23
Medullated fibres (%)	45.39 ${}^{a}$ ± 9.57	73.93 ± 10.09	86.91 ${}^{b}$ ± 7.23
Continuous medullated fibres (%)	10.98 ± 3.80	17.46 ± 4.01	19.73 ± 2.88
Discontinuous medullated fibres (%)	34.41 ${}^{a}$ ± 8.55	56.48 ± 9.01	67.18 ${}^{b}$ ± 6.46

${}^{a,b}$ Different superscripts in the same row represent significant differences between the ages of the alpacas ( P<0.05 ).

The fibre medullation percentage was lower ( P<0.05 ) in the wool of alpacas aged up to 1 year compared to the wool of alpacas over 4 years. In the wool of alpacas up to 1 year of age, the share of non-medullated fibres was higher ( P<0.05 ); the share of fibres with discontinuous medulla was lower ( P<0.05 ) in comparison to the wool of older alpacas and, especially, in comparison to the wool of alpacas over 4 years old. Differences in the share of continuously medullated fibres in alpaca wool in the examined age groups were not recorded.

Similarly to the analysis of wools depending on the colour, the highest number of non-medullated fibres was found in thinner wools, regardless of age. With the increase in wool thickness, the share of medullated fibres was greater. Within the latter fibres, a discontinuous medullation was dominant (Fig. 2).

## 4 Discussion

In the conducted study, no significant differences in terms of fibre thickness and medullation characteristics depending on the colour variety were recorded in the wool of alpacas, although better values of these parameters were found in white alpaca wool (Table 2). The lack of differences in the fibre diameter and degree of medullation depending on the colour variety was also indicated by Wuliji et al. (2000) in a study of Huacaya alpaca wool and by Wurzinger et al. (2006) in a study of llama wool. On the other hand, in earlier studies of the wool of alpacas kept in Poland, it was shown that alpacas with a light colour (white and light beige) were characterized by thinner wool (23.45 vs. 27.16) compared to dark (black and brown) alpacas (Radzik-Rant and Wiercińska, 2021). The aforementioned authors also recorded, similarly to the present study but in the absence of significant differences, that dark wool was characterized by a higher degree of medullation, with a greater share of discontinuous fibres and a much lower share of non-medullation fibres. Both this research and the above-mentioned authors do not confirm the results of McGregor's (2006) study, which indicate greater medullation in alpacas with white wool. McColl et al. (2004) also indicated white wool to be the thinnest in the group of light wools. A lower thickness of light fibres in alpacas bred in the USA was obtained by Lupton et al. (2006). Research conducted on the Peruvian Huacaya alpacas showed the smallest fibre diameter from white alpacas, greater thickness from animals with a cream shade, and the thickest wool from dark ones (Cruz et al., 2017). The relationship between colour and MFD and its variability (CV) was also determined by Canaza-Cayo et al. (2022).

Despite the discrepancies in the results of the research conducted by the above-mentioned authors, it can be seen that dark alpaca wool has worse parameters in terms of fibre diameter and degree of medullation. This may be due to the lack of breeding work in terms of wool quality in dark-coloured individuals due to the fact that, for a long time since, white wools have been preferred by the textile industry (Bustinza and Apaza, 1990).

The most important feature of alpaca wool, which is the average fibre diameter, as well as the content of medullated fibres in it, is undoubtedly influenced by the age of the animals. According to McGregor (2006), the thickness of the coat increases with age. The author believes that the first coat has the best parameters, while, after reaching the age of 6 months to 7 years, the thickness may change by up to 15 µ m. In turn, Hoffman (2006) reports that the average thickness increases until the age of 6 and then decreases. In this study, the difference in the mean fibre diameter between alpacas aged up to 1 year and those aged 1 to 4 years and aged over 4 years was 5.96 and 6.05 µ m, respectively (Table 3). As in the case of these studies, increases in wool thickness by 3.71 µ m and 4.52 µ m in Huacaya and Suri alpacas aged 3 to 9 years were recorded by Cruz et al. (2017). According to the authors, the diameter of the fibres is linear throughout the early years and then tends to stabilize.

Alpaca wool in the group aged up to 1 year showed the best values in terms of average fibre diameter, but the CV MFD value was much higher ( P<0.05 ) than in the wool of older alpacas. In the study of Peruvian Huacaya alpacas conducted by Paucar-Chanca et al. (2019), the wool from 2-year-old animals was thinner than that from the older ones, but no differences in terms of variation (CV) in this feature were recorded. On the other hand, Canaza-Cayo et al. (2022) indicated a large individual variability in terms of CV MFD in the study of the wool of young alpacas (1 year old). The high variability in terms of fibre thickness in young alpacas obtained in this study may be related to the development and maturation of primary and secondary hair follicles.

The age-related follicular maturation and the ratio of primary to secondary hair follicles also affect the frequency of medullated fibres (Antonini et al., 2004). The above-mentioned authors state that the medullation of the fibres increases with age, which was also confirmed in the conducted research. The degree of medullation in the wool of alpacas aged over 4 years increased by over 40 % in relation to the wool of alpacas aged up to 1 year. Differences in the percentage of medullation between alpacas aged 1 to 4 years and those older than 4 years were not observed (Table 3). Therefore, the breeding value of alpacas should be evaluated at the age of 2 years (Vásquez et al., 2015). With age, it was mainly the share of discontinuous fibres that changed. The presence of medullated fibres is associated with a prickling sensation in finished products due to their physicochemical properties (Frank, 2008).

In all tested wool samples, regardless of the average fibre diameter, non-medullated fibres, fibres with discontinuous medulla, and fibres with continuous medulla were found. Medullated fibres were present in samples with a thickness even below 15 µ m, regardless of colour and age (Figs. 1 and 2). The presence of the medulla in very thin fibres was also recorded in studies of Huacaya alpaca wool in Peru and Poland by Piranes et al. (2018), Radzik-Rant and Wiercińska (2021), and Radzik-Rant et al. (2021) using the micro-projection method to measure wool thickness. A greater proportion of medullated fibres was found in thicker wools (Figs. 1 and 2). Identification of fibre types, thanks to the method of determination, indicated the dominance of fibres with a discontinuous medulla. This may give us a chance to eliminate the medullation of alpaca wool faster through selection, which requires consideration, apart from the thickness and degree of the fibre medullation, which are other factors influencing these basic quality characteristics of wool.

## 5 Conclusions

The colour of the wool did not significantly affect the thickness and degree of medullation of the alpaca wool. The observed insignificantly better values of these features in white wool may result from less intensive breeding work and a smaller number of animals with a colourful coat, in addition to the fact that herd owners sell animals to other countries with a lower breeding value.

With age, the mean fibre diameter increased; the share of non-medullated fibres in the fleece decreased; and the share of medullated fibres, especially those with a discontinuous medulla, increased.

All types of fibres (non-medullated and with continuous and discontinuous medulla) were present in the wool of the examined alpacas, regardless of the thickness and colour of the coat and the age of the animals.

## Data Availability

The datasets used and analysed during this study are available from the corresponding author upon reasonable request.
